# Comparative analysis of fresh food e-commerce brand attitudes based on STM theme model

**DOI:** 10.1371/journal.pone.0282521

**Published:** 2023-03-16

**Authors:** Hongyu Liu

**Affiliations:** College of Economics and Management, Heilongjiang Bayi Agricultural University, Daqing, Heilongjiang, China; Victoria University, AUSTRALIA

## Abstract

The circulation of fresh agricultural products is related to the quality of consumption and agricultural development. This article takes the online reviews of fresh products as the research object and studies the comparative differences of consumer brand perception under the different sentiment and brand source classifications. The study was carried out with the aim to explore the influence mechanism of consumers’ different brand attitudes. Structural Topic Modeling (STM) method was used to classify online reviews for brand perception topics, and Gephi network visualization was used to analyze the influence relationship between different brand perception topics. The study also conducts comparative research on the differences in perceived attitudes between positive and negative emotion classifications, as well as self-operated and non-self-operated brands, and analyzes the moderating effect of sentiment scores on the perceived theme intensity of different brands.

## Introduction and literature review

The circulation of fresh agricultural products has attracted intense attention from scholars and business managers in recent years. China Central Government report 2021 once again proposed e-commerce as an important means for the effective connection of production and consumption of agricultural products from villages to cities (China’s "No. 1 central document" for 2021). According to one another survey, the mainstream platform-based e-commerce sales accounted for 74.8% of the fresh food e-commerce market in China, still occupying a dominant position in 2020 (Research Report on China’s fresh e-commerce industry in 2020, iResearch). This means that cooperation with platform e-commerce is considered the best choices sometimes for many fresh suppliers [1, 2].

Another fact is that China’s total import and export of fresh agricultural products across borders was 743-billion-yuan, accounting for 43.5% of the total import and export of agricultural products in 2020, and this status quo has continued to grow rapidly in 2021. Domestic fresh agricultural products occupy a large market share in the national agricultural import and export trade. With the improvement of residents’ living standards, consumers’ preference for imported fresh agricultural products is gradually increasing [3]. However, due to the increasingly fierce competition in the fresh e-commerce market, industrial integration and channel monopoly, e-commerce dividends have not been distributed fairly and effectively. In addition, China has strengthened the regulation of e-commerce platforms. All this increases the risk and uncertainty of cooperation with e-commerce platform and at same time many new companies are facing a dilemma between online or offline sales during recent years [4].

In the context of the upgrading of the consumption structure, the quality and branding of agricultural products has become a new growth point in consumer demand. In present time e-commerce of agricultural products has gradually dependence on brands. It means that new enterprises should build a competitive attractive brand as the main path for e-commerce operation [5]. Main different between traditional offline model of buyer and seller is a more comprehensive setup e-commerce companies to have multiple identities such as platform construction and management and product sales, forming a ternary governance situation with consumers and third-party sellers. So, there are different brand types which are self-operated brand from platform and non-self-operated brand from the third-party sellers [6, 7].

The development of e-commerce has promoted the transformation of traditional agricultural product brokers (vendors) and many agricultural product producers into local e-commerce operators. The non-self-operated brand mainly operated by agricultural product brokers, or by farmers themselves. Due to all these it’s a big challenge for new companies to present multi-agent and complexity of network governance [8]. This phenomenon has led to the confusion for new product suppliers for choosing in way of e-commerce brand agent, self-operated-brand or non-self-operated brand [9], and how to cooperate with them. Ultimately, brand value is determined by consumer attitudes, not brand agents. So deeply mining consumers’ attitude is basically most important for brand development, which is the key for the farmers and also for many new products suppliers, especially for these foreign suppliers. There is no sufficient literate on this problem, there are few researches applied comparative analysis method to study brand attitude. Customers reviews and online comments always play a vital role for other people to decide and buy same brands items [10]. Consumers’ subjective perception of product image and quality is an important part of brand value, an important basis for analyzing and discovering consumer attitudes and challenging corporate brand strategies. So, the researcher can say online feedback is always an important method for research and business management [11]. How to effectively implement the brand strategy and coordinate the multi-agent governance relationship with the platform has become the key to solve the problem of high cost and high risk of fresh e-commerce.

Due to importance of online business, it is getting importance for online foods business as well. The theoretical research on fresh food e-business mainly focuses on fresh food logistics and operation models. To distribute fresh food, it depends on cold chain, city distribution, Internet of Things, communication technology, so scholars explore fresh food storage and transportation losses, logistics costs, distribution efficiency and other issues to solve strategies to improve fresh food logistics service capabilities.

Zan (2020) annotated that the low level of standardization of fresh produce has resulted in an imbalance between quality and purchase costs, making it difficult to build fresh brands, and proposed that companies need to identify consumers’ preference for product characteristics and formulate product quality control Standard building brand elements [12]. But relevant theoretical studies mostly focus on improving the quality of logistics cold chain to solve the problem, while research on e-business brand building pays more attention to product quality and logistics service quality, but the brand connotation in the e-commerce environment, related to quality, price, logistics, online shopping experience and other content. Therefore, digging out consumer online reviews is important for brand management and research of fresh food e-commerce means [13, 14]. Many other researchers used customers feedbacks as a variable to analyze customers attitudes which help them for corporate strategic decision making [15, 16]. In recent years, many studies have adopted unsupervised methods such as topic models and cluster analysis to mine online review texts and extract product features that consumers care about [17]. Tirunillai (2014) applied the LDA theme model to construct a dimensional index for brand evaluation [18]. Gensler (2015) et al. based on the association network model as a theoretical framework, proposed a combination of text mining and network analysis methods, and extracted consumer brand association keywords and their interconnections from online product review texts to form a representative product-brand-memory-network, so that managers can effectively evaluate the advantages and disadvantages of brand image [19].

**Fig 1:**
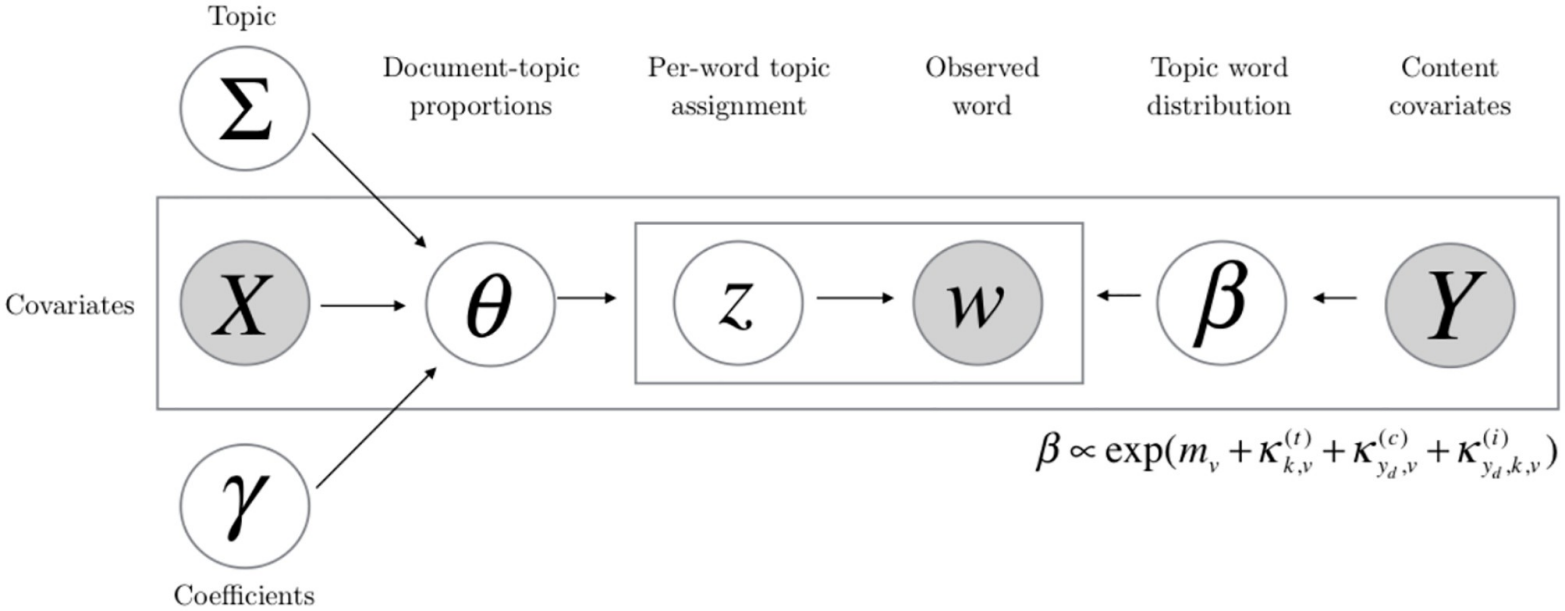
STM structural topic (adopted by Roberts et al., 2016).

This research chosen the STM model to classify and compare the topic of fresh food e-commerce review text. In the research method of topic classification model, the existing research mainly focuses on the topic classification model of LDA (Implied Dirichlet Distribution) [20]. LDA and STM are both Bayesian generative topic models. They assume that each topic is a distribution function of terms, and each document is a mixture of topics in the entire corpus (Fig 1) [21].

After carefully literature review, it is estimated that there is a need of comprehensive research on brand management of agricultural products, especially for fresh food e-business and brand strategies. Therefore, from the perspective of fresh food e-commerce brand building, this article takes consumer brand attitudes as the research object, through text mining on consumer online product reviews, compares the differences in consumer brand attitudes under different categories, and identifies fresh food e-commerce consumption preference characteristics, and then dig out the business strategy of brand growth and value appreciation of fresh e-commerce products. However, existing related research has not yet made a comparative analysis of consumers’ different sentiment and attitude differences under different brand sources. In order to deepen related theories, this paper applies the STM structure topic model to make up for the lack of research methods in this field, and classifies different sentiment. More specifically this research carried out with these research objectives,

1. To study and analyze deep relevant theoretical research to carry out targeted and in-depth research.
2. To use online customers feedback to classify the online reviews of online fresh food consumers, through comparative method study.
3. To analyze why there exists consumer attitudes difference, and analyze the relationship between the consumers’ attitudes, thereby propose fresh food e-commerce brand management and development strategies.
4. To promote the progress of fresh cold chain logistics and promoted the rapid development of fresh food e-commerce.

## Methodology

### STM Topic Model

Comparing to LDA model, the advantage of STM is that it introduces document-level metadata as covariates (covariates can be user data and sentiment associated with a comment from the metadata. Classification, purchase data, etc.) to explain topic strength (topic prevalence, the proportion of topics in each document) and topic content (topic content, topic term distribution), that is, topic prevalence and topic content are specified as any number of metadata covariates [22]. That is, topic prevalence and topic content are specified as a logistic generalized linear model (GLM) with any number of metadata covariates. As the term indicates, GLM is a generalized form of linear regressions. It is additionally adaptable to linear regression because: GLM performs when the output variables are not persistent or unbounded. GLM permits modifications in unconstrained intakes to simulate the output variable on a suitably restrained scale. It helps researchers to adopt STM model to discover the driving factors of user attitudes and the dynamic dependencies between them and output of can be used for hypothesis testing of relationships [23].

The Structural Topic Model (STM) is a form of topic modeling developed explicitly with social science research. STM entitle us to contain metadata in our model and uncover how additional documents might articulate the same underlying topic using various word choices. Latent Dirichlet Allocation (LDA) is an example of a topic model operating to classify a document’s text to a particular topic. It creates a topic per document model and terms per topic model, modeled as Dirichlet distributions.

### Experimental design and data preparation

According to the data and sample, in this study, the researcher chose cherries products as sample, and online consumers’ reviews of cherries products as experimental text data. According to the latest data from the General Administration of customs during 2020, China imported 214,400 tons of cherries (cherry), an increase of 405% over 2012, which is satisfactory data representation. The study applied a crawler program crawled a total of 41234 consumers’ reviews of cherries on JingDong (hereinafter referred to as JD) e-commerce platform from 2019 to 2021. The brand source contained cherries official flagship store which is operated by JD platform, and non-self-operated brand which were selected from top five by sales volume. Data were refined by deleting invalid comments and a total 34,340 were selected for present research. By using the python naive Bayes classifier, the sentiment classification of comments is carried out in the form of supervised learning, including 23329 positive comments and 4661 negative comments. Finally, the author applied RostEA software to calculate the sentiment value separately of the positive and negative reviews sentences one by one, and got the quantitative results of the sentiment value of each review. The RostEA method has been widely used to analyze user text content in Chinese, and the accuracy rate of cross-judgment for the results of the text sentiment mining test has reached 80.6%.

According to the data clean and corpus prepare, R language were used to process the text data, including data segmentation, removal of stop words, and constructing corpus. Step 1: Reviews segmentation. The researcher applied the jiebaR word segmentation package, to segment each review sentence, and eliminated the stop words with two stop words dictionary from Harbin Institute of Technology and Machine Intelligence Laboratory of Sichuan University. Step 2: Building corpus. The researcher applied the corpus generation function prep documents that comes with the STM package to build a document-level corpus of the comment text afterword segmentation.

**Fig 2:**
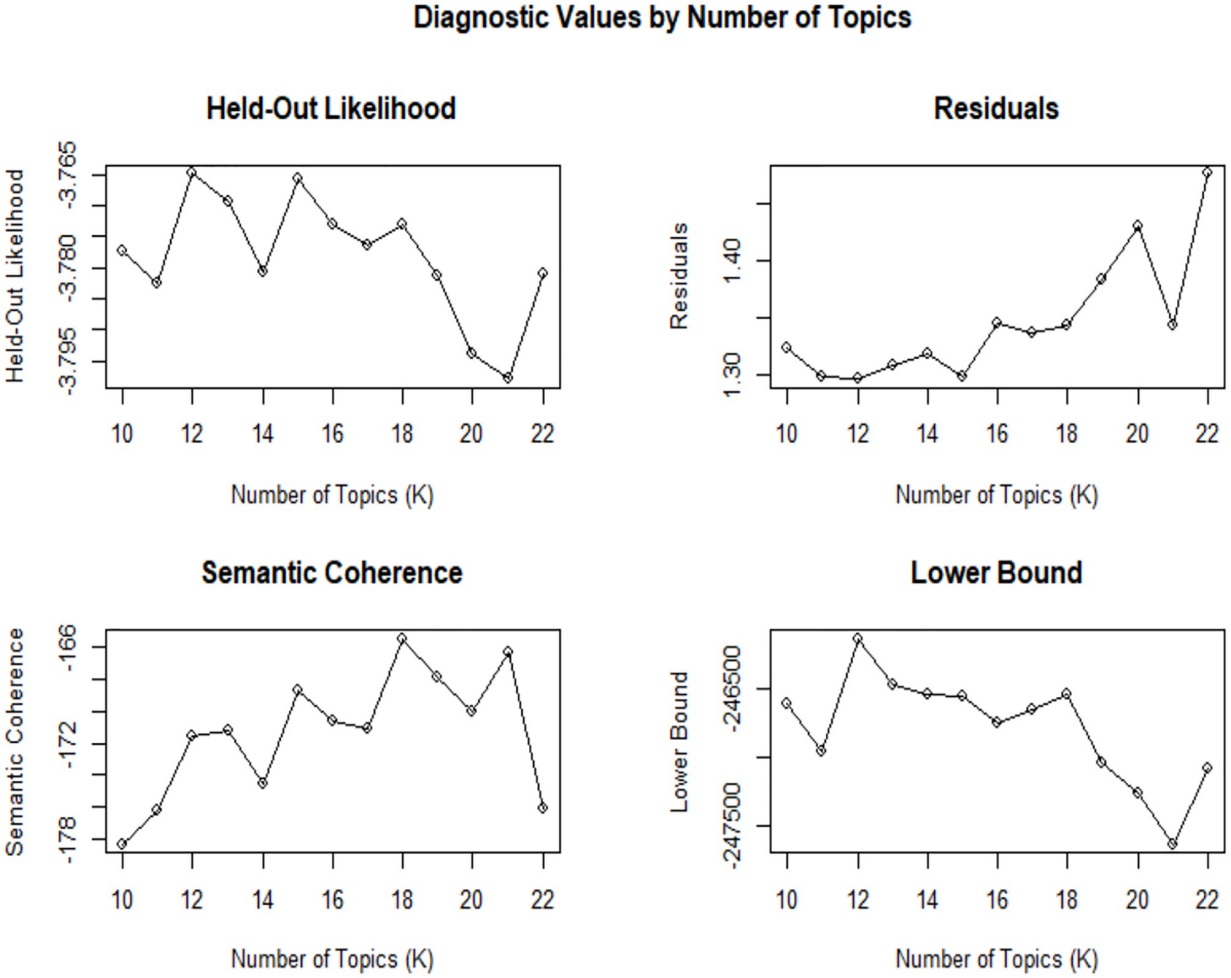
Run results of search K function.

According to calculation for the optimal number of topics, in order to determine the number of topics more accurately, the experiment completed the optimal number of topics (determination of the K value) in 2 steps: Step 1: Diagnostic values. The author applied the search K function of the STM package, set the inspection range from10 to 22 of the K value with 1 as the tolerance, and performed iterative estimation. Roberts et al. (2016) believe that the optimal number of topics can be determined according to four criteria: held-out likelihood, semantic coherence, residual and lower bound. Among them, the first two indicators should be higher, and the latter two indicators should be lower. The experimental calculation results are shown in Fig 2. According to the experimental results, the author found that the fitting effect of 15 to 18 topics numbers was better. Step 2: According to the range of the optimal number of topics determined in step 1, the author ran the model fitting calculations respectively for comparing with k value range from 15 to 18.

According to the covariate variable, the experiment selected 3 covariate variables to construct 2 topic strength calculation models, which are review text sentiment classification (sent.class), brand source classification (source) that include self-operated brand (value 1) and non-self-operated brand (value 2), and sentence average sentiment value (sent.value). The value of sent.class variable range is positive and negative; the sent.value value comes from the calculation result of each comment text by RostEA software, the value range is (-100,100), and the maximum and minimum were deleted. The relationship between Topic Prevalence and sent. class and sent. value as in (1) and (2), where the function g () represents a generalized linear relationship.


Prevalence=g(sent.class,sent.value,sent.class×sent.value) (1)

Prevalence=g(source,sent.value,source×sent.value) (2)

## Results and discussion

### Analysis of results

#### A. Model estimation and results

At first, sentiment value (sent.value) and sentiment classification (sent.class) were used as covariate variables, and input to the STM model according to formula 1 with K range from 15 to 18 to estimate calculation. The STM model was run with the parameters above, and the results are shown in Table 1.

**Table 1:** Topic summary.

**Topic label**	**Top words**
**Class 1**	**product characteristics**
Taste	**Highest Prob**: sweet and sour, sweet, taste, worth, delicious
	**FREX**: taste, sweet and sour, sweet, can’t wait, pregnant
Stale	**Highest Prob**: stale, broken, not tasty, rotten, mostly
	**FREX**: stale, mostly, rotten, soft, broken
Fresh	**Highest Prob**: fresh, no spoilage, refrigerated, tender, elastic
	**FREX**: fresh, refrigerated, granular, color, elastic
Appearance	**Highest Prob**: big, moderate, bright, big, variety
	**FREX**: big, full, moderate, red, full of water
**Class2**	**business services**
Packaging	**Highest Prob**: package, ice bag, fresh, intact, box
	**FREX**: intact, package, tight, ice bag, attentive
Transportation	**Highest Prob**: express, keep fresh, too slow, damaged, refrigerated,
	**FREX**: express, too slow, keep fresh, refrigerate, speed
Communication attitude	**Highest prob**: communication, attitude, consideration, responsible, impatient
	**FREX**: communication, enthusiasm, attitude, impatient, seller
Platform	**Highest Prob**: JD, worth buying, trust, quality, support, self-operated
	**FREX**: trust, support, self-operated, worth buying, guarantee, platform
Promotion Activity	**Highest prob**: price, promotion activity, cost-effective, cheap, catch up
	**FREX**: promotion activity, cheap, catch up, price, second kill
Affordable	**Highest prob**: affordable, cheap, quality, supermarket, quality-price ratio
	**FREX**: supermarket, cheap, affordable, quality-price ratio, discount
Describe truthfully	**Highest Prob**: description, pictures, real objects, advertising, integrity
	**FREX**: description, seller, real object, picture, conform, consistent
After-sales service	**Highest Prob**: seller, bad results, after-sales, compensation, return
	**FREX**: after-sales, compensation, return, seller, rotten
Delivery	**Highest Prob**: fast delivery, express brother, door-to-door delivery, first-class
	**FREX**: fast, express brother, door-to-door delivery, delivery and delivery
**Class 3**	**consumer attitude**
Experience	**Highest prob**: shopping, convenience, experience, door-to-door delivery, happy
	**FREX**: shopping, convenience, experience, door-to-door delivery, convenience
Satisfaction	**Highest Prob**: satisfied, liked, praised, worth buying, cost-effective
	**FREX**: satisfied, praised, liked, worth buying, COVID-19
Recommendation	**Highest Prob**: recommend, friends, take photos, buy, reviews
	**FREX**: recommendation, friends, reviews, photos, buy

The third column of Table 1 presents the top words of each topic. The table lists the Highest Prob (highest probability) and FREX terms of each topic, where FREX is the weighted and average value of semantic coherence and exclusivity, based on the overall frequency of each term and the weighted calculation results of the rejection of other topics. Then, the author inferred the topic label according to the top words of each line in table. The author compared the results separately with topic numbers ranging from 15 to 18, aimed to observe each topic based on the most discriminating terms and most representative reviews. According to the compared results of the model, 16 topics were selected. The second column of the table shows the label of topics, and the first column shows the calculated topic sequence number. All 16 topics can be divided into three main categories: product features, business services, and consumer attitudes.

**Fig 3:**
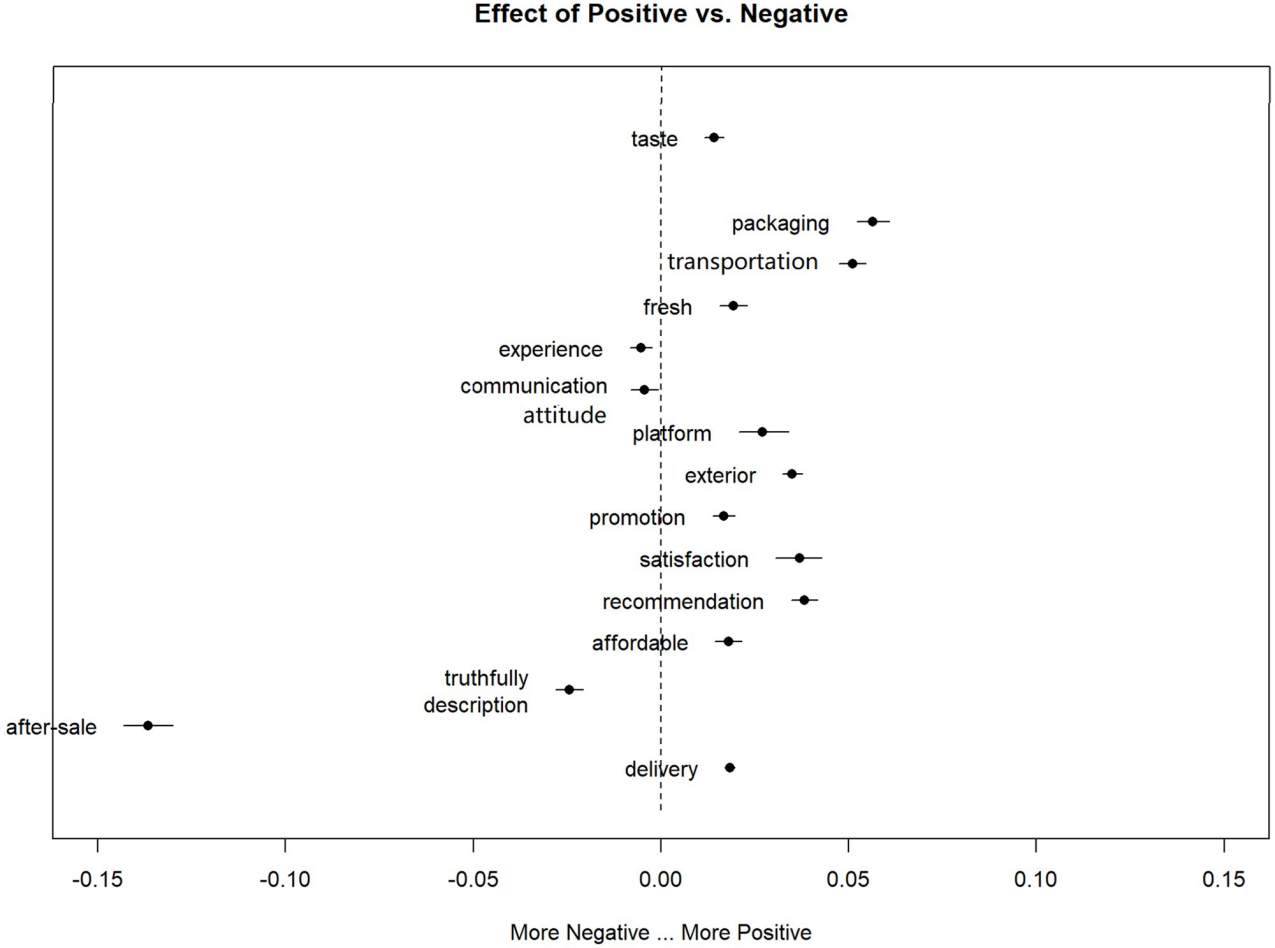
Comparison between different reviews classification.

The STM model was used to calculate the effect of the customers’ feedback classification on the topic prevalence by including the sent.class as a covariate variable in the model, which as a character variable is assigned Positive and Negative by python naive Bayes classifier (Fig 3).

The topic on the left of the dotted line indicates that the proportion of the topic appears in negative feedback is greater than that in positive feedback and vice versa. The author found that experience, communication, after-sale and truthfully description topics appear in the left domain of the Fig 3, which mean they are more likely to appear in the negative feedback. The topic of stale is not on the Fig 3, because the topic proportion value is beyond the boundary -0.15, which mean the topic is the most often appearing in the negative feedback.

Table 1 and Fig 3 can be analyzed that these topics combined to find out which are likely the most complaint by fresh e-commerce consumers. In the communication topic, the author found that the representative words like attitude, considerate, responsible, impatient, mean consumers may often be disappointed by the service staff attitude or willingness. The topic of truthful description tells us consumers are very concerned about whether the information they obtained from webpage pictures or text, or the words of the customer service is true, otherwise they will feel deceived dissatisfaction. Similarly, after-sale service is also one of the concerns of consumers, especially in the process of return and exchange. The author also noticed that the word ‘seller’ appeared in the topic of after-sale and description, that mean consumers have doubt about the products and service capabilities of non-self-brand, compared with self-operated products on e-commerce platforms.

For positive topics, the author can find that consumers have more positive attitude to logistic services like packaging, transportation, delivery and they seem to be willing to express more recognition for e-commerce platforms, such as trust, guarantee etc. For product characteristics, consumers mainly care about appearance, taste, freshness, such as color, granular, sweet, elastic.

**Fig 4:**
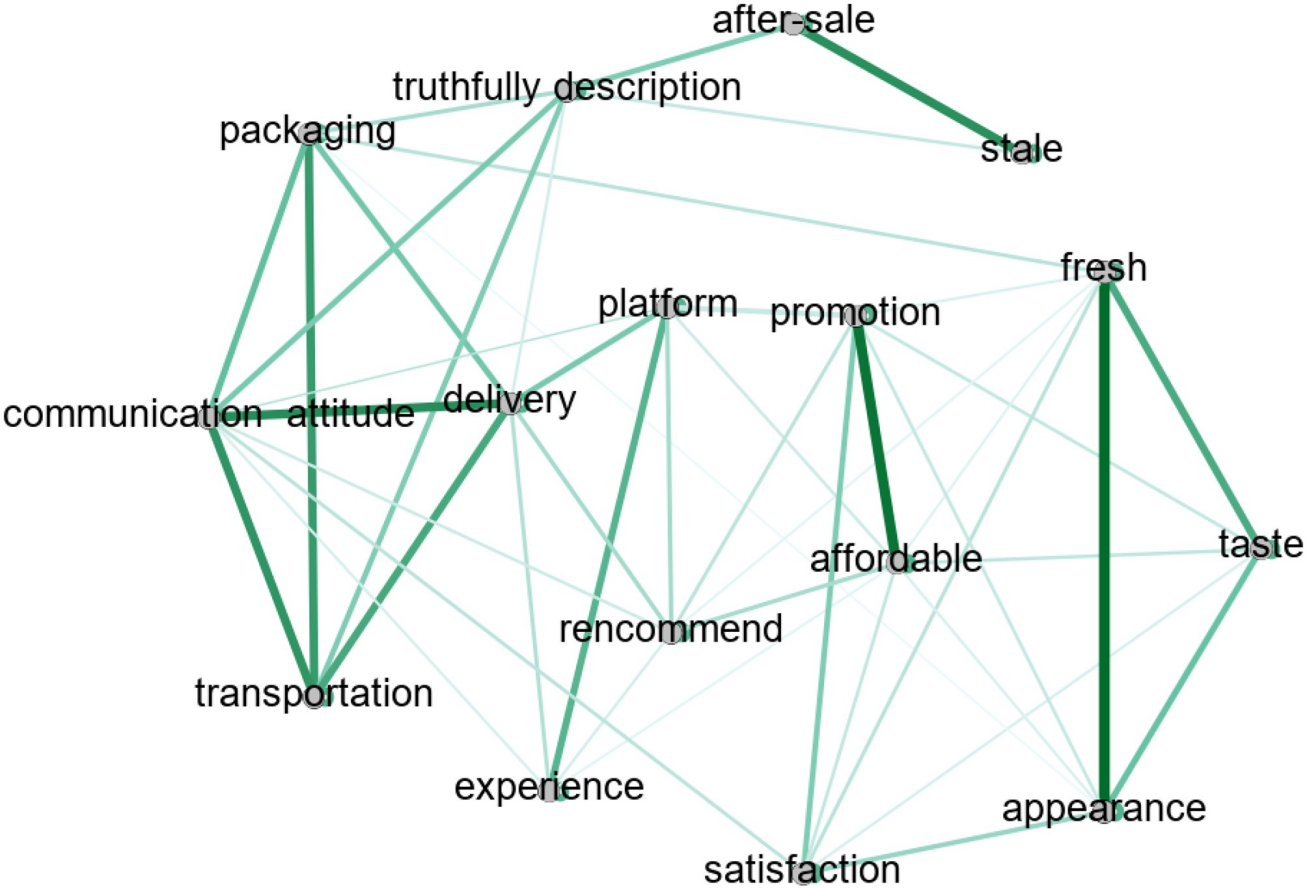
Network of topic correlations.

*Topic correlation analysis*. In order to deeply understand these topics, the author used topic correlation function from the STM package to explicit estimation of the correlation between the topics, which is an important distinctive feature of STM from other topic models. Gephi software was used to generate a network diagram of the correlation between the topics (Fig 4). The nodes in the graph represent topics, the edges between nodes represent the correlation between topics when they tend to co-occur in a piece of feedback, and the color and thickness indicate the strength of the correlation. The darker and thicker of the color, the stronger the correlation. Since the diagram represents the co-occurrence relationship between themes, it helps to understand the potential correlation of consumers’ attitudes.

Fig 4 show explained about consumers’ attitude and it can be found that consumers’ satisfaction is mainly affected by product quality, price and communication, and they prefer to recommend products with high affordable, intact packaging and self-operated brand of e-commerce platform. Communication attitude is a center node that is related with many other nodes. After-sale is mainly related to the stale and truthfully description, and the author can infer consumers may feel cheated for the gap between description and real goods.

At the same time, e-commerce platform has gained more consumers’ positive attitude according to Table 1 and Fig 3, like consumers’ recommendation, experience. One important factor is logistic service capability, for there is a strongly correlation between platform and delivery topic in Fig 4. Promotion may be another factor for many consumers enjoy immersing themselves in shopping festivals in China, which may benefit the e-commerce platform self-operated brand. It can be seen that most fresh e-commerce consumers are price sensitive. This is also the advantage of e-commerce compared to physical stores, especially in China. The self-operated brand of e-commerce companies rely on their stable and mature service quality to ensure a good shopping experience for consumers.

#### B. Comparative analysis of attitudes towards self-operated and non-self-operated brands

**Fig 5:**
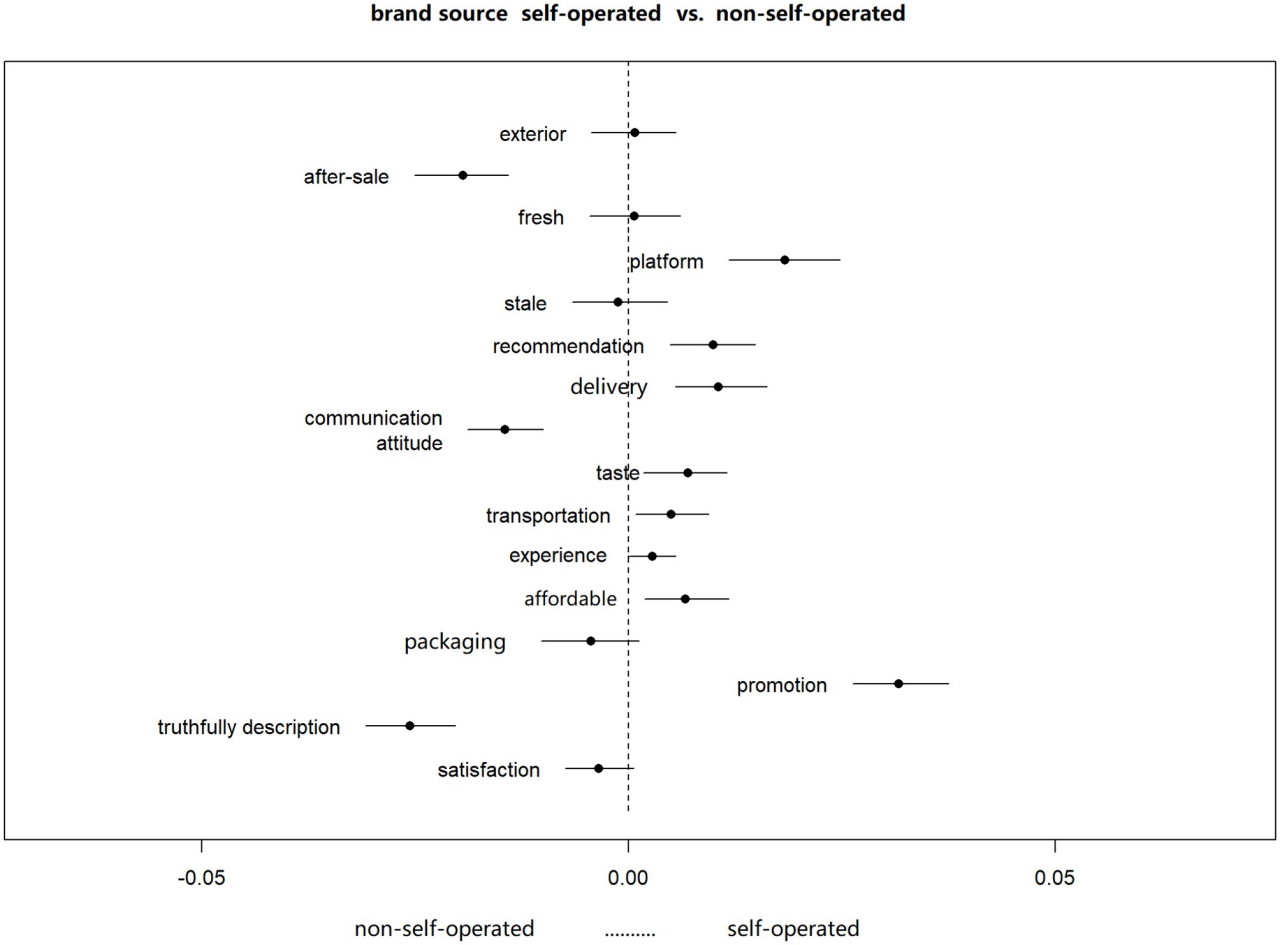
Comparison between different brand source.

Through the above analysis, it can be found that consumers have different attitudes towards self-operated and non-self-operated brands (third-party sellers) on e-commerce platforms. For further analysis, the experiment set the covariates as brand source classification (source) and sentence sentiment value. The sentiment value (sent.value) is brought into the STM model with the K = 16 value, and the topic distribution under the brand source classification (source) is calculated by topic clustering, as shown in Fig 5. The abscissa represents topic prevalence, and the left side of the abscissa is non-self-operated brand, on the right is the self-operated brand. Under this research it found that there is little difference among the topics about product characteristics, such as exterior, fresh, stale. But for business service, consumers’ attitude shows some difference compared different brand sources. For platform self-operated brand, *delivery*, *transportation*, *affordable* and *promotion* topics occurrence more frequently, but for non-self-operated brand, *after-sale*, *communication attitude*, *truthfully description* and *packaging* often appear.

To continue analysis the relationship between business service topics, the topic prevalence was checked. In this part, to compare the effect of business service between the self-operated and non-self-operated brands, so 8 business service topics are selected except platform topic (Table 2). Before estimation, the author standardized the value of variable sent.value.

**Table 2:** Relationship between topic prevalence and sent.value under different topics.

Topic	variable	Estimate	Std. Error	t value	Pr(>|t|)
Packaging	sent.value	-0.0017199	0.0006945	-2.476	0.0133 *
Transportation	sent.value	-0.0015885	0.0005542	-2.867	0.00415 **
Communication attitude	sent.value	0.0027635	0.0007008	3.943	8.06e-05 ***
Promotion	sent.value	-9.25E-04	5.320e-04	-0.179	0.858
Affordable	sent.value	0.0020806	0.0005889	3.533027679	<2e-16 ***
Truthfully describe	sent.value	0.0059680	0.0005438	-10.974	<2e-16 ***
After-sale	sent.value	-0.0097236	0.0007011	-13.87	<2e-16 ***
delivery	sent.value	-0.0022404	0.0002564	-8.738	3.11e-16 ***

Annotation: Signif. codes: 0 ‘***’, 0.001 ‘**’, 0.01 ‘*’, 0.05 ‘.’, 0.1 ‘ ‘

First, the author estimated the relationship between review sentiment value (sent.value) and topic prevalence with regression analysis and it can be seen that the relationship under sales-promotion topic is not significant, and other topics are significant. Second, the author estimated the moderating effect of sentiment value, and the STM model allows us for interactions between the two covariates (source and sent.value).

**Fig 6:**
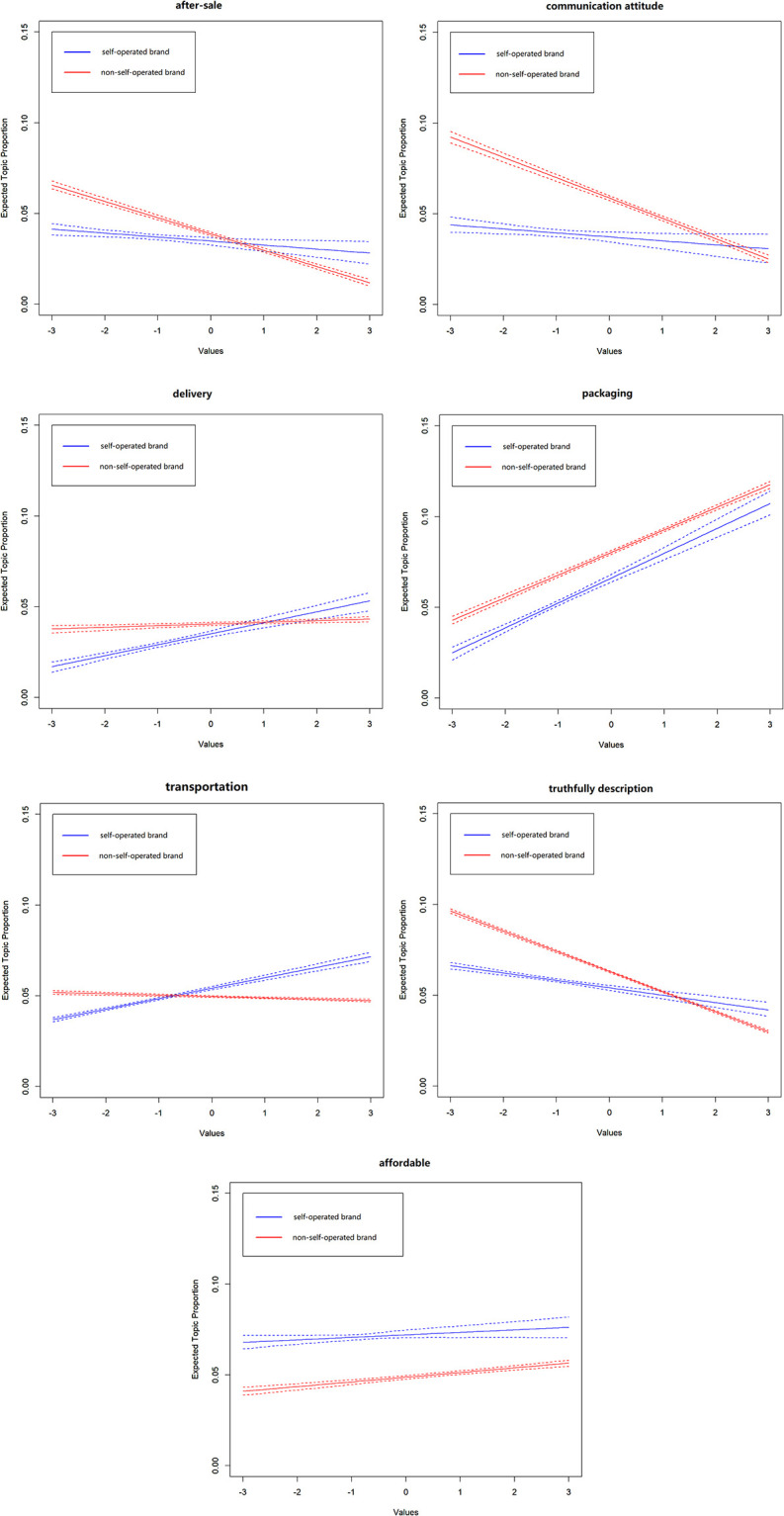
Moderating effects of review sentiment value.

Fig 6 shows the change of the topics proportion, which belongs to business service, with respect to the sentiment value, where the x-axis represents sentiment value and y-axis represents the topic proportion. The non-self-operated brand is shown with red lines, and the self-operated brand is with blue lines. In addition, the dotted lines represent the 95% confidence interval of the estimated value of the subject proportion.

According to Table 2, there are seven topics are shown in Fig 6, which are *after-sale*, *communication attitude*, *delivery*, *packaging*, *transportation*, *truthfully description*, *affordable*. The moderating effect analysis reflects the changes of sent.value and topic prevalence of the seven topics related to business services under two brand sources. The author can find that *after-sale*, *truthfully description*, and *communication attitude* basically exhibit the same variation characteristics. When the sent.value is less than zero, the redlines is higher than the blue, and the sent.value less the redline higher. That means consumers complain more about after-sale, communication attitude and description for the non-self-operated brand compared with the platform self-operated brand.

For delivery and transportation topics, the self-operated brand has received more positive evaluation of consumers compared with the non-self-operated brand, but for *packaging* both of them have the same change curves. It can be found that logistic service, that self-operated brand depending on, from the e-commerce platform has more advantage on fast response and convenient delivery service, comparing the third party logistic which non-self-operated brand usually cooperates with. For packaging, both of the two brand sources have the same service degree, such as using strong packing materials, adding ice packing. It can also find that logistic service can improve consumers’ positive attitude from the blue line rising with the sent.value becomes bigger. But the red line doesn’t rise, which means non-self-operated brand seller should improve their logistic service or strengthen cooperation with the third part logistics. The same condition occurs in the topic of affordable, and the author thinks the self-operated brand is more reasonable in product quality and pricing, also more promotion activities make its product more attractive, which can really improve consumer attitude.

## Conclusions and implications

This paper applies the STM structure topic model to conduct text mining on fresh food e-commerce user reviews, and analyzes the consumption of fresh food e-commerce through topic classification, correlation analysis and moderating effect analysis with the perspective of fresh food e-commerce brand growth. To perceive to conduct subject classification research. When policymakers generate value generation and job development in dynamic sectors, it limits their ability to sustain digital entrepreneurs and tech start-ups. It eventually leads to profits of competitiveness for regional businesses. The paper further explores the characteristics of consumer demand preferences, and the results help companies identify brand competitiveness elements under fresh food e-commerce, rationally formulate brand marketing and development strategies, and further study platform corporate brands and third-party sellers through thematic comparison analysis Brands’ consumer attitudes are different, and the research results help third-party fresh food e-commerce sellers to effectively identify environmental risks and opportunities. According to the research results, particularly in the context of the current e-commerce platform’s leading agricultural Internet upgrade, it will be helpful to the platform enterprises and the third-party sellers to build a more cooperative and sustainable symbiotic relationship. However, this study chooses a single platform and online feedback of a single category of fresh products as the data source, leading to certain limitations in the experimental research results. In the future, horizontal comparison studies can be conducted on different platforms or different categories of products and at the same time, future research can be based on brand competition from the perspective of strength, quantitatively evaluate the competitiveness of different fresh food e-commerce brands, to further explore the influence mechanism of fresh brand competitiveness. These are implications of the present study.

1. E-commerce platform companies need to further coordinate and optimize their relationships with third-party sellers to form a benign competition and cooperation status. Through the above comparative analysis, consumers have doubts about the quality of non-self-operated brand, and to some extent, they trust platform self-operated brand, especially in the after-sales links such as return and exchange of fresh products, platform-operated brands have more advantages. However, this competitive situation is not conducive to the construction and development of non-self-operated brand. Especially in the disadvantaged items of third-party sellers, platform companies can provide cooperation in after-sales service, return and exchange, and build a more open and inclusive empowerment platform for fresh food companies, making self-operated brand and non-self-operated brand form a benign competition and cooperation relationship.
2. Non-self-operated brand sellers need to focus on highlighting the elements of brand personality. Compared with platform self-operated brand, third-party sellers have a lot of space in shaping the personality of non-self-operated brand, such as personalized packaging and product portfolios. Platform self-operated brand, due to its mature operating system and standardized operating procedures, is likely to cause consumers to generate brand trust, but they lack the elements of brand personality, and this area can become a source of competitive advantage for non-self-operated brand.
3. The construction of fresh food brands in the e-commerce environment should not only focus on product quality but also provide good service. A good shopping experience can promote more repurchase and recommendation willingness of consumers, which is an important condition for establishing brand trust. The entire experience of all links after consumption, including delivery, packaging, logistics service quality, customer service communication, return and exchange, etc., and even e-commerce entertainment, which means that companies not only need to control product quality, but also need to improve comprehensive e-commerce service capabilities, and many fresh food companies tend to ignore the improvement of e-commerce service capabilities in their transformational operations under the e-commerce environment.
4. The theme of *affordable* and *promotion* reflects that low-price promotion strategies are effective for fresh price-sensitive products. The pursuit of affordable is an important motivation for consumers to abandon offline consumption and switch to online shopping. The e-commerce circulation model can reduce the operating costs of intermediate links, but there is still a high degree of uncertainty in the conversion of cost advantages to price advantages. Especially between the platform self-operated brand and non-self-operated brand, there is a certain degree of homogenous competition, resulting in the lack of obvious price advantages for third-party sellers. Third-party sellers can adopt a relatively flexible price strategy to avoid price wars with platform brands and affect brand image.
5. Fresh food companies should use online customer’s feedback to establish stable and benign communication channels with consumers. Through the collection of review texts, it can be found that there are a large number of invalid and low-value reviews in online reviews, and companies need to encourage consumers to provide more effective feedback reviews, such as allowing consumers to give corresponding thematic classification feedback according to the theme model. Online reviews not only contain a lot of business value information, but also an important way to build a company-consumer brand relationship. Many consumers’ dissatisfaction attitudes can be effectively handled through timely communication.

## Supporting information

S1 File (DOCX)
