# Supplementary material for: Comparative analysis of fresh food e-commerce brand attitudes based on STM theme model
Source: PLoS One. 2023 Mar 16;18(3):e0282521. doi: 10.1371/journal.pone.0282521 (PMC10019611; doi:10.1371/journal.pone.0282521)
Supplement: S1 File — (DOCX) [file pone.0282521.s001.docx]

## Appendix A: STM text mining code

library(stm)

library(quanteda)

library(readtext)

library(tm)

library(jiebaR)

library(jiebaRD)

cutter <- worker(stop_word = "dic/stop_words.UTF8",output = "script/")

mydata <- read.csv("data/reviewment_all.csv",stringsAsFactors = FALSE, header = TRUE)

content_all <- as.list(mydata$content)

list_all <- list()

for(i in 1:length(content_all))

list_all[[i]] <- segment(content_all[[i]],cutter)

for(i in 1:length(list_all))

list_all[[i]] <- subset(list_all[[i]], nchar(as.character(list_all[[i]]))>1)

corpus_all <- VCorpus(VectorSource(list_all),readerControl = list(language="UTF-8"))

#将语料库保存为txt,并按序列命名语料???

?writeCorpus()

#readtext读取文档

DATA_DIR <- system.file("D://", package = "readtext")

d <- readtext(paste0(DATA_DIR,"/corpus/*"))

#构建quanteda级corpus语料???

data_all <- quanteda::corpus(corpus_all,split_context = FALSE)

#语料库输出保???

write.csv(data_all,file = "data/Vcorpus_all.csv",fileEncoding = "UTF-8")

##dfm包构建dtm矩阵--------------------------------------------------

#对语料库进行分词处理，fastword-按照分隔符分???

toks_data_all <- tokens(data_all,what = "fasterword")

#构建dfm文档词项矩阵

dfm_data_all <- dfm(toks_data_all)

##stm包构建dtm矩阵--------------------------------------------------

#stm模型拟合

poliblogPrevFit1 <- stm(documents = out1$documents, vocab = out1$vocab, K = 21,

prevalence =~source+sentence.sent.value,

max.em.its = 70, data = out1$meta, init.type = "Spectral")

poliblogPrevFit <- stm(documents = out$documents, vocab = out$vocab, K = 18,

prevalence =~class+out$meta$sentence.Likert.value,

max.em.its = 75, data = out$meta, init.type = "Spectral")

interactionfit1 <- stm(documents = out1$documents, vocab = out1$vocab, K=17,

prevalence = ~source*sentence.sent.value,

max.em.its = 75, data=out1$meta, init.type = "Spectral")

interactionfit <- stm(documents = out$documents, vocab = out$vocab, K=18,

prevalence = ~out$meta$class+out$meta$sentence.Likert.value,

max.em.its = 90, data=out$meta, init.type = "Spectral")

cclabelTopics(poliblogPrevFit,topics = 1:30,n=10)

labelTopics(poliblogPrevFit1,topics = 1:30,n=10)

labelTopics(interactionfit,topics = 1:21,n=7)

labelTopics(poliblogContent,topics = 1:11)

topicQuality(poliblogPrevFit,documents = docs,M=10)

plot(poliblogPrevFit,type = "summary")

thoughts3 <- findThoughts(poliblogPrevFit, texts = shortdoc,n = 2, topics = 3)$docs[[1]]

#selectmodels主题一致性和独特性计???

poliblogSelect <- selectModel(out$documents, out$vocab, K = 15,

prevalence =~class+sentence.senti.value,

data = out$meta, runs = 13, seed = 165)

mult <- multiSTM(poliblogSelect, mass.threshold = .75, reg.formula = ~ treatment,

metadata = gadarian)

plot(mult)

plotModels(poliblogSelect, pch=14,legend.position="bottomright")

#输出主题关键???

thoughts3 <- findThoughts(poliblogPrevFit, n = 2, topics = 3)$docs[[1]]

thoughts20 <- findThoughts(poliblogPrevFit, n = 2, topics = 8)$docs[[1]]

par(mfrow = c(1, 2),mar = c(.5, .5, 1, .5))

plotQuote(thoughts3, width = 1, main = "Topic 3")

plotQuote(thoughts20, width = 1, main = "Topic 9")

#相关性分???

sour.factor <- as.factor(sour)

prep<-estimateEffect(1:18~class+sentence.Likert.value,poliblogPrevFit,metadata= out$meta, uncertainty = "Global")

summary(prep, topics=c(3,5,7,11,14,15,16,18))

interaction_est <- estimateEffect(c(1)~class*sent.value, interactionfit,

metadata = out$meta, uncertainty = "None")

plot(interaction_est, covariate = "sent.value", model = interactionfit,

method = "continuous", xlab = "Values", moderator = "class",

moderator.value = "Positive", linecol = "blue", ylim = c(0, .3),

printlegend = F)

par(new=TRUE)

plot(interaction_est, covariate = "sent.value", model = interactionfit,

method = "continuous", xlab = "Values", moderator = "class",

moderator.value = "Negative", linecol = "red", ylim = c(0, .3),

printlegend = F)

legend(-80, .2, c("Postive", "Negative"),lwd = 2, col = c("blue", "red"))

for(i in 1:18){

interaction_est <- estimateEffect(c(i)~class*sentence.Likert.value, interactionfit,

metadata = out$meta, uncertainty = "None")

plot(interaction_est, covariate = "sentence.Likert.value", model = interactionfit,

method = "continuous", xlab = "Values", moderator = "class",

moderator.value = "Positive", linecol = "blue", ylim = c(0, .2),

printlegend = F, main = paste("Topic",i,sep = " "))

par(new=TRUE)

plot(interaction_est, covariate = "sentence.Likert.value", model = interactionfit,

method = "continuous", xlab = "Values", moderator = "class",

moderator.value = "Negative", linecol = "red", ylim = c(0, .2),

printlegend = F)

legend(-80, .2, c("Postive", "Negative"),lwd = 2, col = c("blue", "red"), title = "topic")

}

plot(prep,out$meta$sentence.senti.value,method = "continuous")

#不同主题的词???

poliblogContent <- stm(out$documents, out$vocab, K = 8,

prevalence =~source+sent.value,

content = ~source, max.em.its = 75,

data = out$meta, init.type = "Spectral")

poliblogContent <- stm(out$documents, out$vocab, K = 16,

prevalence =~class+sentence.sent.value,

content = ~class, max.em.its = 75,

data = out$meta, init.type = "Spectral")

plot(prep, covariate = "class", topics = c(1,2,3,4,5,6,7,8,9,11,12,13,14,15,16,18),

model = poliblogPrevFit, method = "difference",

cov.value1 = "Positive", cov.value2 = "Negative",

xlab = "More Negative ... More Positive",

main = "Effect of Positive vs. Negative",

xlim = c(-.15, .15), labeltype = "custom",

custom.labels = c('taste','stale','packaging','transportation','fresh',

'experience','communication attitude','platform','exterior',

'promotion',

'satisfaction','recommendation','affordable',

'truthfully description','after-sale','delivery'))

plot(prep, covariate = "class", topics = 3,

model = poliblogPrevFit, method = "difference",

cov.value1 = "Positive", cov.value2 = "Negative",

xlab = "More Negative ... More Positive",

main = "Effect of Positive vs. Negative",

xlim = c(-.1, .1))

plot(prep, covariate = "source", topics = 8,

model = poliblogPrevFit, method = "difference",

cov.value1 = "JD", cov.value2 = "THD",

xlab = "More Negative ... More Positive",

main = "Effect of Positive vs. Negative",

xlim = c(-.1, .1))

mod.out.corr <- topicCorr(poliblogPrevFit)

write.csv(mod.out.corr[["poscor"]], file = "mod.out202112.csv")

plot(poliblogContent, type = "perspectives", topics = 10)

plot(poliblogPrevFit, type = "summary", topics = 1:16)

plot(mod.out.corr)

#igraph 可视化输???

library(igraph)

library(psych)

igraph = graph_from_adjacency_matrix(

mod.out.corr$poscor,mode="undirected",weighted=TRUE,diag=FALSE)

fc = cluster_fast_greedy(igraph,weights =NULL)# cluster_walktrap cluster_edge_betweenness, cluster_fast_greedy, cluster_spinglass

modularity = modularity(igraph,membership(fc))

# 按照模块为节点配???

comps = membership(fc)

colbar = rainbow(max(comps))

V(igraph)$color = colbar[comps]

set.seed(123)

plot(igraph,main="Co-occurrence network",vertex.frame.color=NA,vertex.label=NA,

edge.lty=1,edge.curved=TRUE,margin=c(0,0,0,0))

set.seed(123)

plot(igraph,main="Co-occurrence network",edge.width=1,

vertex.size=5,edge.lty=1,edge.curved=TRUE,margin=c(0,0,0,0))

plot(igraph,main="Co-occurrence network",layout=layout_with_kk,

vertex.frame.color=NA,vertex.label=NA, edge.lty=1,

edge.curved=TRUE,margin=c(0,0,0,0))
